# Virtual environments as memory training devices in navigational tasks for older adults

**DOI:** 10.1038/s41598-018-29029-x

**Published:** 2018-07-17

**Authors:** Ismini E. Lokka, Arzu Çöltekin, Jan Wiener, Sara I. Fabrikant, Christina Röcke

**Affiliations:** 1 University of Zurich, Department of Geography, Zurich, 8057 Switzerland; 2 Bournemouth University, Department of Psychology, Bournemouth, BH12 5BB UK; 3 University of Zurich, University Research Priority Program (URPP) “Dynamics of Healthy Aging”, Zurich, 8050 Switzerland

## Abstract

Cognitive training approaches using virtual environments (VEs) might counter age-related visuospatial memory decline and associated difficulties in wayfinding. However, the effects of the visual design of a VE in route learning are not fully understood. Therefore, we created a custom-designed VE optimized for route learning, with adjusted levels of realism and highlighted landmark locations (MixedVE). Herein we tested participants’ route recall performance in identifying direction of turn at the intersection with this MixedVE against two baseline alternatives (AbstractVE, RealisticVE). An older *vs*. a younger group solved the tasks in two stages (immediate *vs*. delayed recall by one week). Our results demonstrate that the MixedVE facilitates better recall accuracy than the other two VEs for both age groups. Importantly, this pattern persists a week later. Additionally, our older participants were mostly overconfident in their route recall performance, but the MixedVE moderated this potentially detrimental overconfidence. Before the experiment, participants clearly preferred the RealisticVE, whereas after the experiment, most of the younger, and many of the older participants, preferred the MixedVE. Taken together, our findings provide insights into the importance of tailoring visualization design in route learning with VEs. Furthermore, we demonstrate the great potential of the MixedVE and by extension, of similar VEs as memory training devices for route learning, especially for older participants.

## Introduction

Navigation is a key component of human daily life, both when moving between locations in familiar environments, and when reaching new destinations in unfamiliar environments. Especially in unfamiliar environments, navigation can be a difficult task. Because of the age-related decline of some of the perceptual and cognitive abilities that support navigation, this difficulty increases as people age^1,2^. In this paper, we seek to develop a better understanding of difficulties and facilitators in route learning for older adults. Specifically, we examine the potential of virtual environments (VEs) that are custom-designed for route learning in compensating for age-related decline in navigational skills.

There are numerous technology-driven approaches to assist with wayfinding, and many dedicated devices provide real-time navigation instructions such as mobile phone apps or in-car navigation devices^3^. These devices assist people in navigating in the real world, but they are not necessarily optimized for *learning* novel routes or entire environments^4^. In fact, the real-time assistance might contribute to the decline in the ability to independently navigate, as a large portion of the mental effort is externalized to the device and no active engagement from the user is necessary^5^. This argument would be in line with cognitive aging propositions of “use it or lose it”^6^. In this context, we view VEs as candidate visuospatial *memory training devices*. Such a memory training device might benefit everyone, but might be especially meaningful for those who struggle to remember routes when walking in unfamiliar environments, such as is often the case for older adults^7,8^. Training can lead to improvements in the trained domain, such as spatial abilities^9^, route learning performance^10–12^, as well as other cognitive skills^13–16^. VEs are widely used for navigation-related cognitive training^17^, as they provide a safe, controlled substitute for the real world. As such, user errors pose no harm to people while navigating, display design can be personalized, and one can navigate the virtual route as many times as needed without too much physical effort. VEs, however, also possess various limitations. Importantly, most VEs only provide visual stimulation. Other sensory information typically involved in locomotion in the real world, such as vestibular-, proprioceptive-, and efferent-information, are reduced or non-existent in most VEs^18^. The VE setups that stimulate senses other than vision remain complex to set up, and can be prohibitively expensive^19^. Additionally, linked to the decades-old “experimental control vs. ecological validity” debate^20^, one might question whether the learning that occurs in a VE is applicable in the real world, even though a VE has the advantage to simulate the real world to a larger degree than other laboratory-based cognitive training experiments with very abstract stimuli. We believe VEs are promising memory training devices, because the benefits we listed earlier and the advantages it offers over other training methods outweigh their limitations. Besides, previous research presents evidence that learning with tools (maps, VEs) might be applicable in the real world^17^. Importantly, and in contrast to the real world, VEs can be easily and systematically manipulated, which allows addressing research questions that cannot be addressed in real world settings^21^. For example, by highlighting or suppressing certain visual features in a VE, we can investigate which visual information is most relevant for the memorability of a route, thus informing the development of navigational training tools in the future.

A common proposition found in visualization literature is that the more closely the representation of an object resembles its real-world counterpart, the easier it is to relate to it, and people are therefore more likely to remember it^22–24^. In contrast, realism in visuospatial displays impairs performance in certain spatial tasks, including the memorization of a route from 2D maps due to factors such as cognitive load^25–27^. It is also important to note that, unaware of their impaired performance in perceptual tasks, people consider realistic visualizations attractive, and thus might be misguided in their visualization preferences^28^. This concept is coined as ‘naïve realism’, and it appears to be particularly relevant for people with lower spatial abilities who do not calibrate their preferences even after working with the visualizations that are better for them (people with higher spatial abilities do)^28^. As spatial abilities decline in older age^29^, naïve realism might be an important concept to consider when studying how older adults interact with visualizations.

The potential of VEs as memory training devices for older adults in the context of route learning, and the effects of varying the design of VEs (specifically, optimizing the realism levels and landmark locations) on the memorability of the routes, are poorly understood^7^. Here we address this gap as it has important consequences for the development and design of novel interventions to target the highly relevant ability of successful navigation, and thus independent living. In the following sections, we review the key literature regarding memory decline in older adults, particularly in a navigation context; and investigate the potential of VEs as memory training devices from a visualization design perspective.

## Navigation in Older Adults: Remembering, Forgetting, and Training

As mentioned previously, it has been well-documented that aging has a negative effect on navigation performance^7^. Especially in unfamiliar environments, older adults experience greater navigational difficulties than younger adults^7,8,30^. Such difficulties can discourage older adults from exploring new environments, and negatively affect their independence and overall quality of life^31^. These age-related navigation difficulties derive from a decline in the relevant visuospatial abilities and memory capacity, both of which vary widely across individuals^32^. Most memory systems, including visuospatial memory that is necessary for navigation, seem to weaken across the lifespan^33^; and this has been documented both in virtual and real world experiments^34–38^. As memory declines, people make more misattribution errors^39,40^, that is, an actual experience of an event may be misplaced in time, place or source when retrieved from memory^41,42^. Misattribution errors are common amongst older adults, especially when there are many things to remember^41^. Additionally, it has been shown that older adults overestimate the accuracy of their memories and they are too confident on specific details of their recent experiences^43^. However, the findings on misattribution errors and memory-related overconfidence in older adults might be context dependent. Existing studies are often limited to memorizing lists of words^44^, and in certain cases, to presenting pictures and videos, such as videos of crime-scenes^45,46^. When it comes to route learning in unfamiliar environments, it has been shown that older adults tend to confuse the location of landmarks at critical decision points^47^. At this point, however, we know little about the effects of visual design on misattribution errors and memory-related overconfidence. Thus, identifying the optimal design choices for the features of visuospatial training material that facilitates navigational performance in later life seems warranted.

## Visuospatial Displays as Training Devices for Route Learning

Learning is the consequence of a complex interplay between sensation, perception, cognition, and experience^48^. In many learning tasks, visuospatial information processing plays a key role. A number of design decisions on how the visuospatial information is represented might affect memory, and consequently, impair or improve route learning^49–51^. We find that a VE optimized for route learning should be balanced for the *amount*, the *quality* and the *position* of the presented information^52^. Quality related considerations are beyond the scope of this paper^52,53^. In this paper, we focus on the amount and the position of the presented information, and we examine their impact on route recall *in combination* (i.e., not independently).

Cognitive load is one of the strongest arguments against visual realism as a display principle^28^. Controlling for the *amount* of information by varying the levels of realism is one way to address cognitive load in route learning in a VE. Depending on the context, one can also highlight landmarks by using symbols (e.g., arrows, letters, colors, outlining the object) or by placing discrete objects at critical locations serving as landmarks, and it has been shown that such approaches increase their memorability^54^. On the other hand, as mentioned earlier, an important argument in favor of realism is that a high degree of realism might make it easier for people to recognize, name and thus relate to the elements (e.g., trees, benches, windows) on a display^22^ as they acquire a meaning^24^. ‘Nameability’ of items might be helpful in memorizing them, for example, people remember nameable colors better than others^55^. It has been proposed that the verbal memory systems help in such cases, because people do not rely *only* on visuospatial memory systems for key executive functions such as the encoding, storage and recall of information (i.e., the dual channel theory)^56^. However, in learning from visualizations, the question of ‘how much information is too much/too little?’ remains persistent^57,58^. In the case of a VE, one might use photo-textures selectively to maintain a sense of realism, and to enable recognition of features, while reducing cognitive load at the same time. On the other hand, a certain level of abstraction guides the attention to task-relevant features^27^, which might facilitate remembering and learning.

Besides the amount of information, the *position* of a feature within the scene imposes an important consideration for route learning. Navigation studies and landmark theories mention some unambiguously relevant visuospatial elements that are positioned in specific places in the visual scene for route learning^59–63^: Structural, visual, and/or semantic features determine the importance of landmarks^64–69^. Specifically, *decision points* are critical as in these points people ‘take mental notes’ of a feature and retain that as a landmark; and reportedly, these features are consistently located in the direction of turn^60^. Related to the position of features, or classes of features, it appears that the structural network (i.e., street network and its spatial layout) provides another important visual anchor in route learning, and might contribute to the memorability of a scene^70^.

## Our Study

Synthesizing previous work summarized above, we designed an ‘optimized VE’ for route learning, which we call the MixedVE. The VEs in this study were named based on their relation to abstraction-and-realism; however, note that the optimization is based on *two* important considerations; (a) reducing the *level of realism* by removing photo-textures from task-irrelevant parts of the VE (i.e., manipulating the *quantity* of visual information), and (b) deliberately choosing the locations of the photo-textured elements (i.e., manipulating the *position* of visual information). Because we are interested in *optimizing* the MixedVE as a memory training device, we combined these considerations when designing the MixedVE. Also note that, while we do not investigate aspects of *quality* in this paper, we counterbalance the content of the textures for their semantic qualities based on a previous qualitative assessment for their levels of memorability^52,53^.

**Figure 1:**
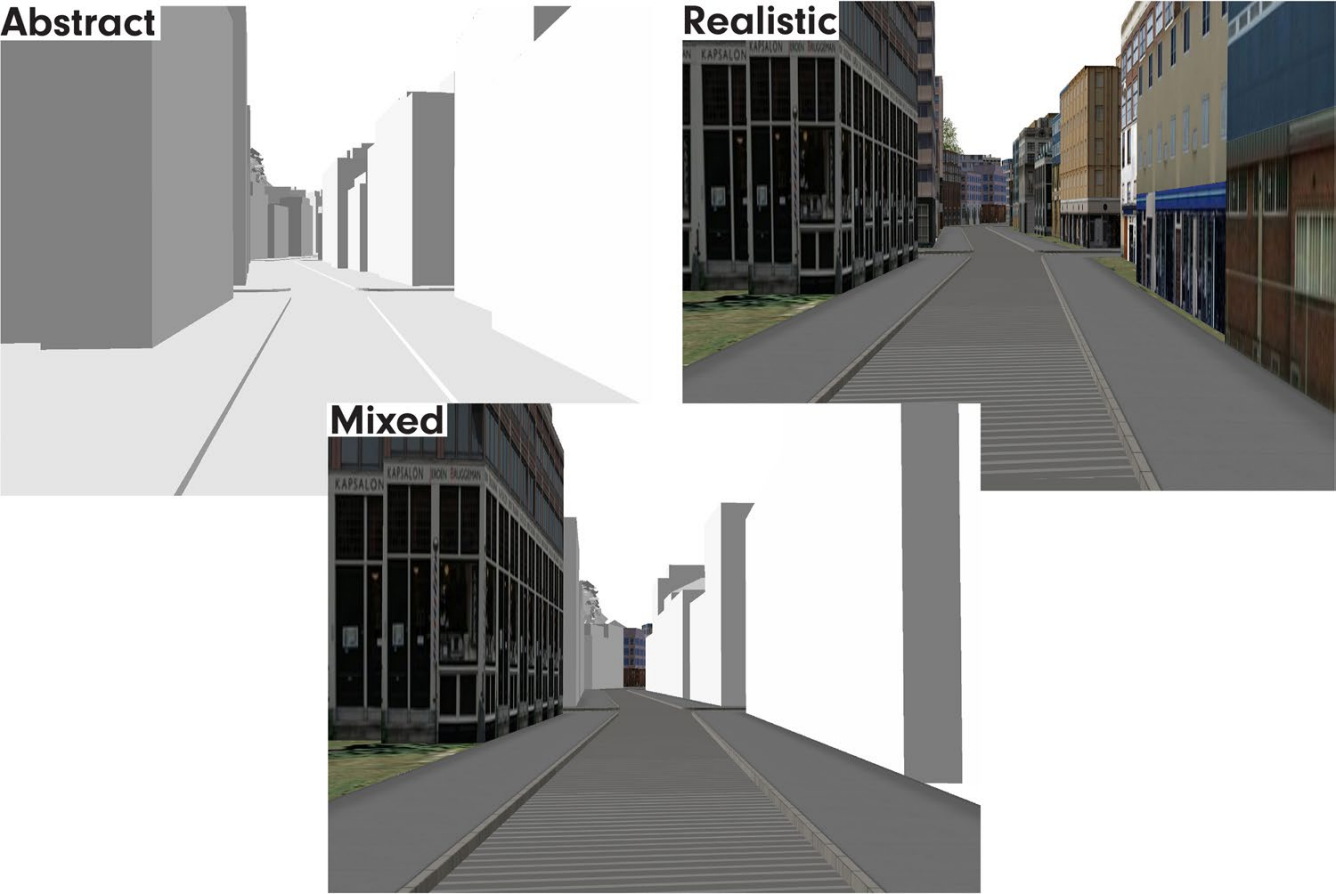
Screenshots from the three VEs to illustrate their visual designs (not to scale). The AbstractVE is rendered in grayscale without photo-textures, whereas the RealisticVE is fully photo-textured. The MixedVE is a combination of the two in which most elements are rendered in grayscale but buildings at critical positions and the road network are photo-textured.

In sum, in the MixedVE, we highlight selected elements in the scene (i.e., buildings at decision points positioned in the direction of the turn along the route of interest, and the street network) with realistic photo-textures, and suppress the rest by removing photo-textures. Figure 1 shows an illustration of the MixedVE and the other two VEs we used for comparison (AbstractVE and RealisticVE). We chose to compare the MixedVE with a RealisticVE as a high-fidelity representation of the real world. The RealisticVE contains all the visual information including the photo-textures at the navigation-relevant scene elements, however, it does not highlight the navigationally relevant environmental features. The AbstractVE, on the other hand, serves as a baseline condition with no photographic information, and again, no highlighting effect. The fact that the AbstractVE contains considerably less information should significantly reduce the cognitive load induced by photo-textures, although it might increase task difficulty otherwise, because of the lack of anchor points.

We previously demonstrated that younger adults overall benefit from the MixedVE compared to the AbstractVE and RealisticVEs in visual, spatial, and visuospatial memory tasks in a route learning context^53^. In this paper, we examine the potential of the MixedVE as a memory training device in route learning, particularly for older people. Our leading hypothesis is that the MixedVE will successfully serve older people as a memory training device in the context of route learning, specifically, in memorizing and identifying the direction of turns at the intersections, because of the following sub-hypotheses:

- Due to the balanced cognitive load and the selective highlighting as a consequence of retaining photo-textures only in navigation-relevant scene elements, irrespective of their age, participants should identify the direction of turn at intersections better (thus, recall the route better) with the MixedVE than with the other VEs, both immediately after the experiment, and a week later.
- Irrespective of age, participants’ overall *confidence* in their responses should better align with their recall accuracy with the MixedVE than other VEs. In addition, overall, *older* participants should be *overconfident* in their responses in comparison to younger participants. Thus, the moderating effect of the MixedVE should be more pronounced for the older participant group.
- *Before* the experiment, both older and younger participants should prefer the RealisticVE^25,28^. *After* the experiment, younger participants should change their preferences to the MixedVE. Older participants, however, due to the decline in some of the relevant spatial abilities, might not be able to identify which visualization supports them better, and thus should still prefer the RealisticVE after the experiment.

We tested our hypotheses in a between-subject experiment with an older group (65–75 yrs.) and a younger group (20–30 yrs.) as a comparison group. In the experiment, participants watched a driving simulation video, in which they viewed the route from the ‘passenger seat’ and were asked to memorize the route. After they watched the videos, participants were given various visuospatial recall tasks in two ‘recall stages’ (immediate vs. delayed by a week) to measure *learning*. In this paper, we focus on one of the task types; that is, identification of heading direction at intersection points. This is a typical task in route learning studies and previous findings allow us to build our age-related hypotheses for this task type^67,71–74^. In the Procedure section, we describe all of the tasks for full disclosure, and elaborate further on our choice on focusing on this task type. We report the main findings for the two other tasks in the *Appendix: Additional Analysis*. Our three independent variables are *visualization type* (the three VEs), *age* (older vs. younger), and *recall stage* (right after the experiment vs. one week later), whereas we measured three dependent variables: participants’ route recall *accuracy*, their *confidence* in their recall performance, and their visualization *preference* before and after the experiment.

## Results

We first report the overall route *recall accuracies* of the younger and older participants (age), for the immediate and delayed recall stages (recall stage) with all three VEs (visualization type). Furthermore, we report the *forgetting rates*, (the difference in recall accuracies between the two recall stages) for both groups. We then analyze participants’ confidence in their responses. Since confidence in one’s success in solving a task can be viewed as one’s “perceived accuracy” on that task; we compare the *perceived* and the *actual* accuracies of participants to examine underconfidence or overconfidence (known as *calibration error*^43^). Last but not least, we present participants’ visualization *preferences*, and how these preferences shifted among the three VEs before and after the experiment.

Sample size has been estimated via a power analysis using the G-power software. In all tests in which significant results were obtained, the F test was followed by Bonferroni’s post-hoc test for multiple comparisons. Associated *p*-values < 0.05 are reported as statistically significant, along with the effect sizes (${\eta }_{p}^{2}$, r, and Cohen’s d). Following the convention, we interpret ${\eta }_{p}^{2}$ values 0.01, 0.06, 0.14; and Cohen’s d values 0.2, 0.5, 0.8; and r values 0.1, 0.3, 0.5 as *small*, *medium* and *large* respectively.

### Recall performance based on age, recall stage, and visualization type

**Figure 2:**
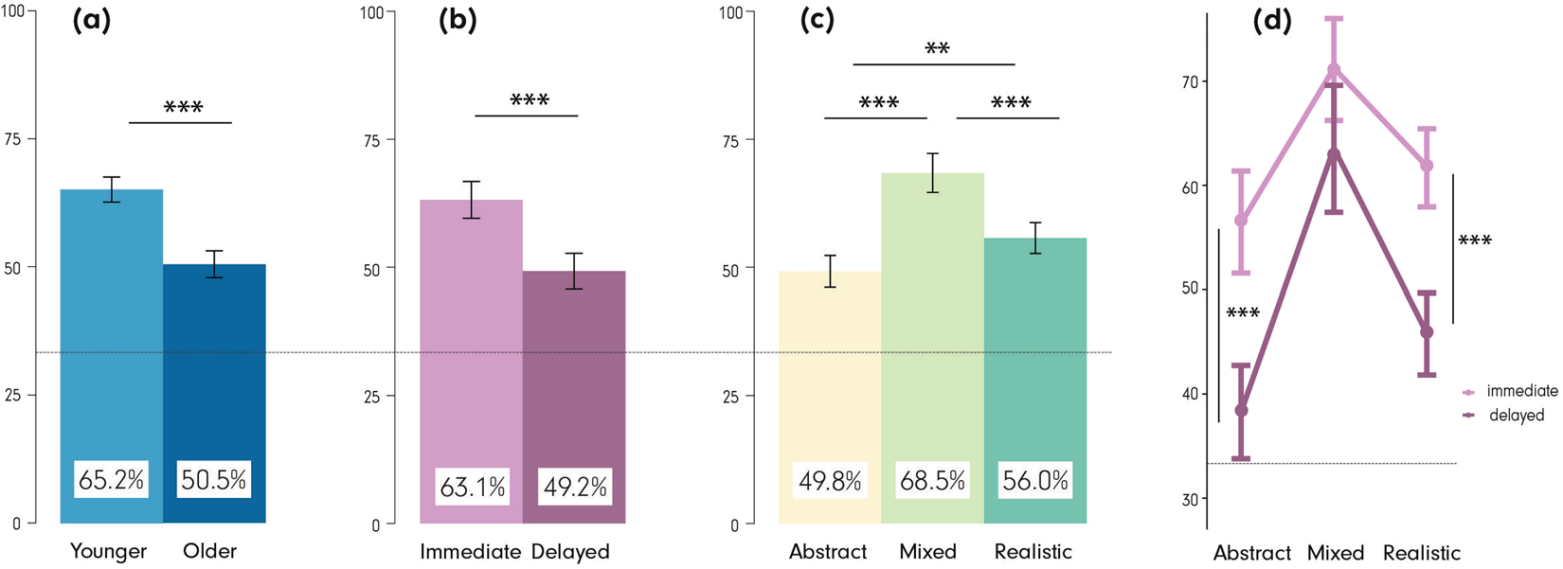
Main effects of (**a**) *age*, (**b**) *recall stage*, and (**c**) *visualization type* on recall accuracy, and (**d**) interactions between *recall stage* × *visualization type* (irrespective of age). The chance level is marked with a light line in 33% recall accuracy. ***p < 0.001, **p < 0.01. Error bars: SEM.

A 2 (age) × 2 (recall stage) × 3 (visualization type) mixed-design ANOVA revealed significant differences in recall accuracies for all three independent variables. Figures 2a–c depict the main effects for: (2a) *age*, F(1, 79) = 29.96, p < 0.001, ${\eta }_{p}^{2}$ = 0.10 (young: 65.2% ± 11.5%, older: 50.5% ± 11.7%); (2b) *recall stage*, F(1, 79) = 46.17, p < 0.001, ${\eta }_{p}^{2}$ = 0.10 (immediate: 63.1% ± 16.6%, delayed: 49.2% ± 16.0%); and (2c) *visualization type*, F(2, 158) = 45.78, p < 0.001, ${\eta }_{p}^{2}$ = 0.14 (AbstractVE: 49.8% ± 17.4%, MixedVE: 68.5% ± 19.3%, RealisticVE: 56.0% ± 16.0%). Pairwise comparisons revealed significant differences in participants’ recall accuracies between the three VEs. Specifically, recall accuracy was higher with the MixedVE than with the Abstract (p < 0.001, d = 1.02) and the Realistic VEs (p < 0.001, d = 0.7), and higher with the RealisticVE than the AbstractVE (p < 0.01, d = 0.37). Furthermore, the *recall stage* × *visualization type* interaction was significant F(2, 158) = 3.73, p = 0.03, ${\eta }_{p}^{2}$ = 0.01 (see Fig. 2d). This interaction was driven by greater and statistically significant performance declines between the immediate and delayed recall with the Abstract (18.0%; t(159.32) = 5.31, p < 0.001, r = 0.38) and the Realistic VEs (16.0%, t(159.93) = 5.42, p < 0.001, r = 0.39), while performance decline in the Mixed VE was smaller and non-significant (7.7%; t(154.26) = 1.89, p > 0.05, r = 0.15). None of the other interactions rendered significant results.

**Figure 3:**
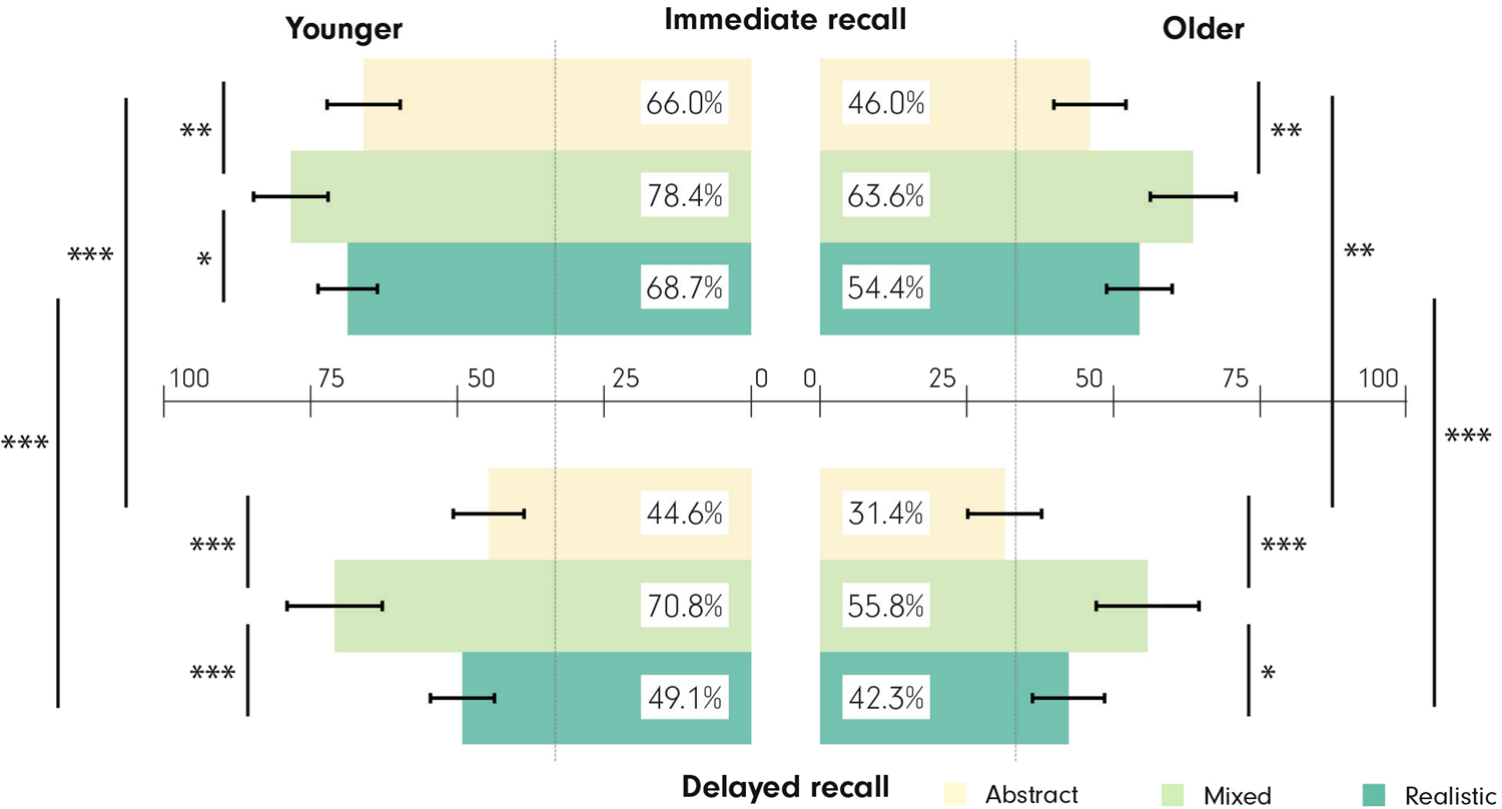
An overview of participants’ recall accuracies in the experimental tasks organized by *age*, and *recall stage* for the three visualizations. Left: Younger participants, Right: Older participants. Top: Immediate recall stage, Bottom: Delayed recall stage. The chance level is marked with a light line in 33% recall accuracy. ***p < 0.001, **p < 0.01, *p < 0.05. Error bars: SEM.

Even though we did not observe interactions between *age* × *recall stage* × *visualization type* F(1, 79) = 0.58, p > 0.05, ${\eta }_{p}^{2}$ = 0.00, we present an overview of the relative recall accuracies of the two age groups in the two stages in Fig. 3. This is accompanied with the inferential statistics in Table 1, to demonstrate how the MixedVE facilitates recall performance better than other VEs in *all* conditions.

**Table 1:** Differences in participants’ recall accuracies.

Age	Recall stage	Repeated measures ANOVA	Pairwise comparison
Younger	Immediate	F(2,84) = 7.80, p < 0.001, ${\eta }_{p}^{2}$ = 0.07	M-A p < 0.01**, d = 0.60 M-R p = 0.02*, d = 0.51 R-A p > 0.05, d = 0.15
	Delayed	F(2,84) = 20.3, p < 0.001, ${\eta }_{p}^{2}$ = 0.22	M-A p = 0.001***, d = 1.11 M-R p < 0.001***, d = 0.95 R-A p > 0.05, d = 0.24
	Immediate-Delayed (forgetting rate)	(*only pairwise*)	AllVE: t(41) = 5.36, p < 0.001***, r = 0.64 A: t(41) = 5.49, p < 0.001***, r = 0.65 M: t(41) = 1.33, p > 0.05, r = 0.20 R: t(41) = 5.77, p < 0.001***, r = 0.67
Older	Immediate	F(2,78) = 9.00, p < 0.001, ${\eta }_{p}^{2}$ = 0.11	M-A p < 0.01**, d = 0.82 M-R p > 0.05, d = 0.44 R-A p > 0.05, d = 0.45
	Delayed	F(2,78) = 13.9, p < 0.001, ${\eta }_{p}^{2}$ = 0.16	M-A p < 0.001***, d = 1.00 M-R p = 0.02*, d = 0.56 R-A p > 0.05, d = 0.55
	Immediate-Delayed (forgetting rate)	(*only pairwise*)	AllVE: t(38) = 4.10, p < 0.001***, r = 0.55 A: t(38) = 2.89, p < 0.01**, r = 0.42 M t(38) = 1.83, p > 0.05, r = 0.28 R: t(38) = 3.85, p < 0.001***, r = 0.53

In the pairwise comparison column, the VE that facilitates the higher recall accuracy is listed first. M:MixedVE, A:AbstractVE, R:RealisticVE. ***p < 0.001, **p < 0.01, *p < 0.05.

### Participants’ confidence in their recall performance

The calibration error was obtained by dividing the recall accuracies by confidence ratings (“perceived accuracies”). For better readability, we scaled the obtained values to diverge from zero, with zero being the perfect match between perceived and actual recall accuracy, and values diverging in opposite directions from zero signifying overconfidence(o) and underconfidence(u).

**Figure 4:**
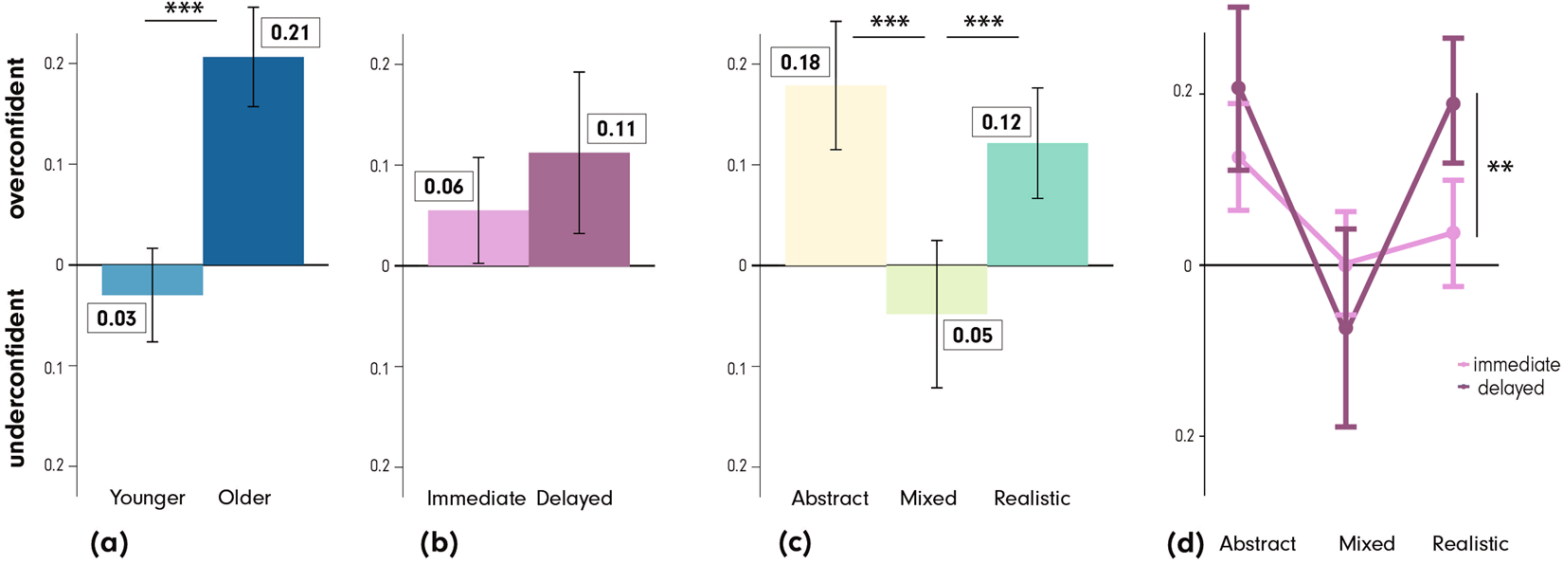
Main effects of (**a**) *age*, (**b**) *recall stage*, and (**c**) *visualization type* on the calibration error, as well as (**d**) interactions between *recall stage* × *visualization type* (irrespective of age). ***p < 0.001, **p < 0.01. Error bars: SEM.

A 2 (age) × 2 (recall stage) × 3 (visualization type) mixed-design ANOVA revealed significant differences in participants’ calibration errors. The main effects are shown in Fig. 4a–c for (4a) *age* F(1, 79) = 23.46, p < 0.001, ${\eta }_{p}^{2}$ = 0.08 (younger: 0.03/u ± 0.43, older: 0.21/o ± 0.39), (4b) *recall stage* where there is no significant effect F(1, 79) = 1.98, p > 0.05, ${\eta }_{p}^{2}$ = 0.01 (immediate: 0.06/o ± 0.32, delayed: 0.11/o ± 0.51), and (4c) *visualization type* F(2, 158) = 18.17, p < 0.001, ${\eta }_{p}^{2}$ = 0.06 (Abstract: 0.18/o ± 0.41, Mixed: 0.05/u ± 0.48, Realistic: 0.12/o ± 0.36). At the visualization level, pairwise comparisons showed that the calibration errors differed between the MixedVE and the AbstractVE (p < 0.001, d = 0.51), as well as between the MixedVE and the RealisticVE (p < 0.001, d = 0.4). Participants are only slightly underconfident with the MixedVE, whereas they are clearly overconfident with the other two VEs. As in recall performance analysis, *recall stage* × *visualization type* interacted: F(2, 158) = 7.13, p < 0.01, ${\eta }_{p}^{2}$ = 0.02 (Fig. 4d); calibration errors with the MixedVE were close to zero in both stages (immediate: 0 ± 0.31, delayed: 0.09/u ± 0.60), whereas participants exhibited overconfidence both with the AbstractVE (immediate: 0.13/o ± 0.32, delayed: 0.22/o ± 0.49) and the RealisticVE (immediate: 0.04/o ± 0.32, delayed: 0.21/o ± 0.38). Pairwise comparisons show that participants were even *more* overconfident with the RealisticVE in the delayed recall stage (t(155.32) = 3.14, p < 0.01, r = 0.24) than in the immediate recall stage. None of the other interactions rendered significant results.

**Figure 5:**
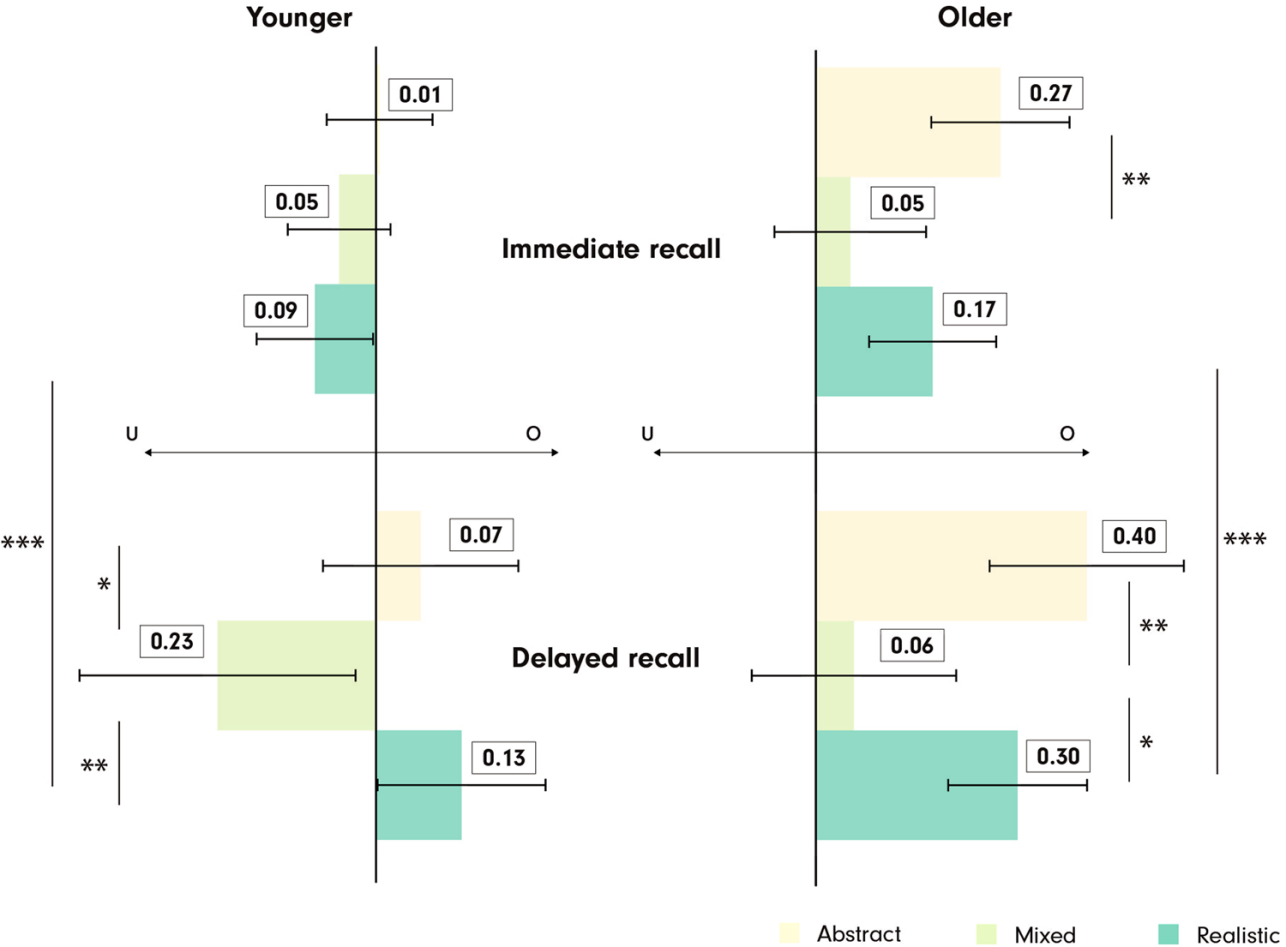
Participants’ calibration errors organized by *visualization type*, *age*, and *recall stage*. Left: Younger participants, Right: Older participants. Top: Calibration errors in the immediate recall stage, Bottom: Calibration errors in the delayed recall stage. ***p < 0.001, **p < 0.01, *p < 0.05. Error bars: SEM. u: underconfidence, o: overconfidence.

Similarly as in the recall accuracy analyses, even though the *age* × *recall stage* × *visualization type* 3-way interaction for the calibration error was not statistically significant F(1, 79) = 1.95, p > 0.05, ${\eta }_{p}^{2}$ = 0.00, we present an exploratory overview of the calibration errors of the two age groups in the two stages in Fig. 5, along with the inferential statistics in Table 2. These results demonstrate that the two age groups may have different calibration error patterns. The younger participants rated themselves relatively accurately (they were slightly underconfident) in the immediate stage with all three VEs. In the delayed stage, the younger participants grew overconfident with the Abstract and Realistic VEs, whereas underconfidence persisted with the MixedVE. The older participants were consistently overconfident in all tested conditions, but clearly with the least calibration errors with the MixedVE (close to zero) in both stages. With the lapse of time, both age groups became significantly overconfident with the RealisticVE.

**Table 2:** Differences in participants’ calibration errors.

Age	Recall stage	Repeated measures ANOVA	Pairwise comparison
Younger	Immediate	F(2,84) = 1.98, p > 0.05, ${\eta }_{p}^{2}$ = 0.02	M-A p > 0.05, d = 0.23 M-R p > 0.05, d = 0.13 R-A p > 0.05, d = 0.35
	Delayed	F(2,84) = 8.01, p < 0.01, ${\eta }_{p}^{2}$ = 0.08	A-M p = 0.04*, d = 0.51 R-M p < 0.01**, d = 0.65 A-R p > 0.05, d = 0.14
	Immediate-Delayed	(*only pairwise*)	A: t(41) = 0.76, p > 0.05, r = 0.12 M: t(41) = 1.54, p > 0.05, r = 0.23 R: t(41) = 3.33, p < 0.01**, r = 0.46
Older	Immediate	F(2,78) = 6.94, p < 0.01, ${\eta }_{p}^{2}$ = 0.07	M-A p < 0.01**, d = 0.65 M-R p > 0.05, d = 0.37 R-A p > 0.05, d = 0.32
	Delayed	F(2,78) = 8.63, p < 0.001, ${\eta }_{p}^{2}$ = 0.10	M-A p < 0.01**, d = 0.73 M-R p = 0.01**, d = 0.59 R-A p > 0.05, d = 0.25
	Immediate-Delayed	(*only pairwise*)	A: t(38) = 1.44, p > 0.05, r = 0.23 M: t(38) = 0.07, p > 0.05, r = 0.01 R: t(38) = 3.00, p < 0.01**, r = 0.44

In the pairwise comparison column, the VE that leads to the least calibration error is listed first. M:MixedVE, A:AbstractVE, R:RealisticVE. ***p < 0.001, **p < 0.01, *p < 0.05.

### Preference for specific visualization types

**Figure 6:**
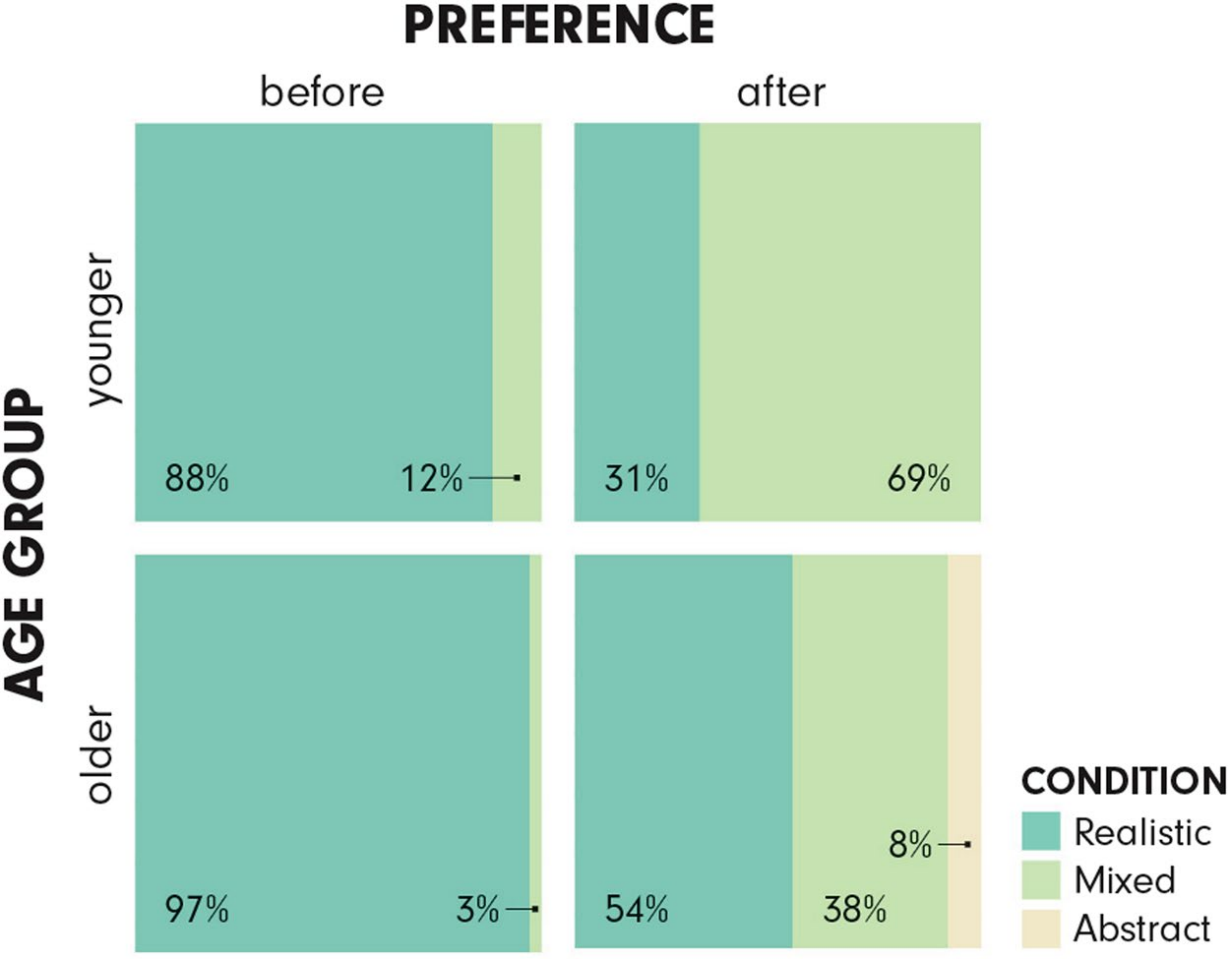
Visualization preferences of younger and older participants before and after the experiment.

Participants’ preferences for the three VEs *before* and *after* the experiment are presented in Fig. 6.

As Fig. 6 shows, *before* the experiment, the younger participants mostly preferred the RealisticVE (88%), while only 12% preferred the MixedVE (*none* prefers the AbstractVE). For the older participants, this is even more pronounced: 97% preferred the RealisticVE and the remaining 3% preferred the MixedVE (again none preferred the AbstractVE). *After* the experiment, however, 69% of the younger participants favored the MixedVE, while 31% kept their initial preference for the RealisticVE (the AbstractVE remains unpopular). Older participants display a different pattern: 54% of them still preferred the RealisticVE, a considerable 38% switched to the MixedVE, and 8% preferred the AbstractVE.

The shift in visualization preference from RealisticVE to MixedVE was statistically significant both for the younger (χ^2^(1) = 67.41, p < 0.001), as well as for the older (χ^2^(1) = 41.86, p < 0.001) participants. No shift from MixedVE to RealisticVE occurred. The odds ratio (i.e., the effect size) of the younger participants changing their preference from RealisticVE to MixedVE were 16.03 (7.440, 37.090), whereas for the older, this was 22.41 (6.635, 119.180). Due to the unpopularity of the AbstractVE (zero values), we did not include it in the chi-square analysis.

## Discussion

Despite the popularity and promise of VEs^17^, little is known about how older adults are affected by differently-designed VEs in route learning tasks in comparison to younger adults. This is surprising given the importance of maintaining spatial functioning and navigational skills to independently conduct daily life activities across the lifespan. Synthesizing knowledge from several disciplines, we designed an experiment to investigate the potential of a custom-designed MixedVE as a memory training device in route learning. We expected that the MixedVE would help all users; however, because of the age-related decline in visuospatial memory capacity, we were particularly interested in the performance of the older participant group. We included a fully photo-textured RealisticVE as the ‘gold standard’ because this is a high-fidelity representation of the real world, and an AbstractVE with no photo-textures as a baseline, to examine if, and how much, our customized MixedVE improves the memorability of the given route in comparison to these two VEs.

### Route recall accuracy improves with the MixedVE irrespective of age

Our findings clearly confirm that the MixedVE improves recall accuracy of all participants in intersection-by-intersection visuospatial route learning tasks (i.e., in identifying direction of turn) considerably and consistently with large effect sizes; both immediately after the experiment, as well as one week later (Fig. 3). Note that these results are consistent across the ‘spatial tasks’ as well, whereas we do not observe a clear pattern for the ‘visual tasks’, possibly because recall accuracies are close to chance level with the visual tasks (see *Appendix: Additional analysis* for overall recall accuracy results based on visual and spatial tasks). Overall, our findings provide clear support for the notion that design decisions are important for successful utilization of VEs for route learning. Note that since we manipulated *two* design elements in the MixedVE — adjusting the level of realism, and deliberately selecting the landmark locations^60^ — we cannot distinguish whether and how much each of the two manipulations influence route recall performance. However, the purpose of the study was not to disentangle the contribution of these two design decisions, but to evaluate an “optimized” design. This required us to consider previous knowledge about what design decisions might improve route learning and recall performance. Our findings suggest that reducing the amount of realism while keeping crucial (i.e., navigationally-relevant) information, indeed assists participants in both age groups in identification of the turn directions, and by extrapolation, route recall in general. In other words, since we were set to measure an optimized design against baseline alternatives, we will not discuss the separate effects of realism levels and landmark locations; these were shown by others in dedicated experiments.

Previous work suggests that visualizations that contain too much or too little information can have negative effects on memory performance^28,58^. Our results *regarding Abstract and RealisticVEs* confirm that both too much and too little information indeed impair performance in a VE-based route learning task, and also importantly, this is true also for older adults (65–75 yrs). As mentioned earlier, another key design decision was the position of the highlighted landmarks in a virtual scene. It is known that people rely on landmarks at specific locations in wayfinding tasks^60,70,75^. Our results *with the MixedVE* suggest that ‘highlighting’ landmarks at task-relevant locations (i.e., in our case, retaining the photo-textures only in navigation-relevant locations) contributes to intersection-by-intersection route memorization and learning in a VE setup where participants learn passively from a video.

It must be noted that, given that we use VEs as a proxy to real world, using photo-textures for highlighting the features of importance is an appropriate choice, and might transfer well to the real world through the resemblance of detail found in photography. However, the use of photo-textures to highlight the selected features (in this case, buildings and the structural network) is one of the many ways one might design a VE as a memory training device. Other means of highlighting, such as using color or outlining the features of interest, may also prove useful. Therefore, it would be useful to examine other means of highlighting in future experiments for a holistic understanding of highlighting techniques for memory training devices. Also note that the decision to remove realistic detail from a virtual scene immediately triggers the question of *where* such removal would be most appropriate. Removing realistic textures randomly (or based on other criteria) might lead to different outcomes than what we observed in our study. Because we aimed to optimize the MixedVE for route learning, we retained the realistic detail at locations that are relevant to route learning, and for the task examined in this paper, our design decisions provided benefits to the participants.

Our main findings in the recall accuracy analyses confirm an age-related difference disfavoring older adults in route learning performance with medium to large effect sizes^7,8,29,30^. Overall, younger participants recalled routes more accurately than the older participants; irrespective of the visualization type and recall stage (Fig. 3). A closer inspection reveals that *age* and *visualization type* do not interact: Recall performance for both older and younger participants were best with the MixedVE, and the two age groups’ recall performance were similar in the two stages. While the age-related memory decline and its various effects on cognitive functions are well documented^47^, studies that examine age differences in connection to levels of realism in visuospatial displays are rare. In this study, we observe that the abundance or lack of visual information do not seem to affect the older group *differently* than the younger. Our findings suggest that the complications linked to “too little” and “too much” visual information are fundamental problems that transcend age-related differences.

In contrast to the other two VEs, recall performance did not significantly decline after a week with the MixedVE for either age group (Fig. 3a). This finding is important, because it suggests that removing unnecessary information from a realistic VE *and* leaving it only in navigation-related locations (compare MixedVE vs. RealisticVE); while highlighting relevant information in navigation related locations — in this case, with realistic photo-textures — (compare MixedVE vs. AbstractVE), support *learning* beyond short-term route memorization.

A surprising finding regarding the two recall stages was that the *forgetting rates* of the older participants were not stronger than those of the younger ones after one week. Thus, our findings in the context of route learning in VEs support the notion that age differences in memory are stronger in *encoding* than in *retrieval*, as our older participants did not necessarily experience problems in retrieving the information (stored in their memory) one week later. Evidence regarding age differences in encoding versus retrieval is mixed, however, current understanding is that both are affected by age. An earlier study that tested memory for positions of the pawns in a chess game^76^ (a visuospatial task at a different scale) also suggested that it is more the encoding than the retrieval process that is affected by aging. Some other studies, carefully designed to tease apart encoding and retrieval processes experimentally, in contrast, have shown that encoding, retrieval, as well as forgetting rates are negatively affected by aging^77–79^. These differences may depend on a multitude of factors, such as the context in which the studies are conducted or the individual differences among the participants. Further research may help understanding such contradicting observations better.

Overall, as both age groups seem to benefit from the MixedVE, we believe that the basic design assumptions of the MixedVE are fitting choices for route learning in VEs, and that MixedVE, and by extension, similarly designed VEs, have clear potential as memory training devices irrespective of age.

### Older participants benefit from the MixedVE in calibrating their confidence

It has been previously shown that older people are overconfident in cued recall tasks unrelated to navigation^43^. Thus, we hypothesized that older participants might also be overconfident in route recall tasks. Our calibration error analysis confirms that older participants indeed overestimate their route recall performance in general, in both the immediate and delayed stages with medium effect sizes. This is somewhat alarming, because in a route learning scenario, arguably, overconfidence can be more of a threat than underconfidence. That is, a false belief that one has ‘learned the route’ might lead to premature action and complications in wayfinding. From this perspective, the fact that the calibration errors with the MixedVE in the delayed recall stage are near-zero for the older group is a very promising result. In other words, with the MixedVE, older people might be less prone to overestimate their performance, and take fewer risks. The younger group is somewhat underconfident with the MixedVE, however, we believe this is less of a threat; as a consequence, they might behave more carefully while navigating after learning with the MixedVE, or practice more.

Both age groups, but particularly the older group seems to be overconfident with the AbstractVE and the RealisticVE in the delayed recall stage with medium effect sizes. With the AbstractVE, the overconfidence may at first appear surprising, as with such low accuracy, one would expect the confidence ratings to be low. Perhaps the visual similarity of the objects to one another, such as it is the case with buildings in the AbstractVE, led to misattribution errors, resulting in a false sense of familiarity a week later when recalling is harder than immediately after the experiment. Similarly, we observe that both age groups had a false belief that they are doing better with the RealisticVE in the delayed recall. This might be explained by the previously documented mismatch in people’s accuracy and confidence in other contexts^80^. In this case, because people could identify particular elements in the visual scene after a week passed, they falsely believed that these assisted them to recall a route. Note that the results regarding the calibration error analysis should be viewed as an exploratory analysis, as the overall interaction of *age* × *visualization* × *recall stage* did not reach significance; while these results allow us to hypothesize, more testing is needed to confirm them.

Overall, the MixedVE afforded a better self-assessment than the other two VEs with medium effect sizes for both age groups, possibly because participants could more precisely recall what they have seen. Importantly, the MixedVE offered a clear advantage for the older group, enabling them to calibrate their confidence that matches their performance much better; thus lending itself as a promising candidate for the development of novel training paradigms for all, but especially for older adults.

### Participants prefer the RealisticVE before the experiment, but many switch to MixedVE after

We find clear signs of naïve realism^28,81^ when participants stated their preferences for the visualization types *before* the experiment. Both age groups overwhelmingly preferred the RealisticVE before the experiment (younger: 88%, older: 97%). These results provide unambiguous evidence of how strongly people are attracted to realistic displays^81^.

*After* participants experienced the VEs and solved the route recall tasks, however, we saw dramatic changes in participants’ preferences. As predicted, most of the younger participants shifted their preference from the RealisticVE to the MixedVE after the experiment. This suggests that the younger participants successfully identified the assistance they received from the MixedVE, and valued their performance with it (i.e., sometimes people prefer the inferior product knowingly, simply because they like it). Nonetheless, a notable sub-group of younger participants (31%) stayed with their original preference for the RealisticVE. The older participants’ preferences after their experience with the VEs show a different pattern than the younger participants’: Even though a large number of older participants also switched to MixedVE (38%), the RealisticVE remained their favorite choice also after the experiment (54%). This may be linked to the overall lower exposure and experience with VE technologies. Furthermore, in Smallman and John’s 2011^81^ naïve realism study, participants with lower spatial abilities did not necessarily change their preference towards less realistic displays after the experiment, even though those with higher spatial abilities did. Perhaps our findings and theirs are linked; one can speculate that people who do not perform too well for various reasons (age or lower spatial abilities) might be less deliberate about the tools they choose. Thus, when designing future visuospatial memory training devices intended for people with limited experience and abilities, it is important to remember that the acceptance of the proposed device might be a barrier to achieving the memory improvement goals, and additional considerations might be necessary.

## Conclusions

Motivated by earlier work on cognitive training, and informed by the principles of visualization design, we tested if one can customize a VE, which could eventually be used as a memory training device in a route learning context. Importantly, because visuospatial memory is negatively affected by age, we focused our efforts on understanding how well our candidate memory training device (the MixedVE) would work for older adults. Specifically, we focused on the visual design of the VE, because design choices can have a strong impact on how well a visualization functions, including its memorability. Thus, we examined aspects of design that should be considered for creating memorable VEs, especially for route learning. Our intuition, as well as the previous work suggested that we represent the world with high fidelity, and replicate the reality in a simulated environment. However, previous empirical evidence in various other contexts led us to believe that we can improve the design of the VE to better function as a memory training device for route learning if we control the amount of visual realism instead. However, we did not ‘randomly’ remove redundant information. Instead, we designed the MixedVE, in which we used photo-textures only at the navigation-relevant locations, that is, where we knew people would look for landmarks. By ‘translating’ the previous empirical evidence into design from two perspectives (realism and landmark use), we essentially highlighted navigation-relevant information in the locations that matter to the viewer to increase their saliency and memorability, and we suppressed less relevant information to reduce cognitive load.

Our results provide new insights for the design of VEs and their possible use as visuospatial cognitive training devices for route learning, especially in older adults. Overall, the MixedVE *was* more memorable than the others, and it facilitated high recall accuracy in identification of turn-of-direction tasks at the intersections (and by extension, in route learning), irrespective of age, both in short and long term. The fact that the MixedVE facilitated both immediate and delayed recall and in both age groups shows how effectively the design choices can improve performance whether one is old or young. Furthermore, the stable recall performance with the MixedVE even a week after the participants watched the simulated video (only once), clearly demonstrated its promise as a potential training device. Participants’ confidence in their performance matched their actual performance better with the MixedVE compared to the other VEs, and this is especially evident for older participants. The fact that the MixedVE helps with adjusting for overconfidence in older adults has important positive implications on their potential navigational behavior. Furthermore, a large number of participants preferred the MixedVE to others after working with it, even though some more design adjustments might be necessary for an older audience.

Taken together, our findings demonstrate the potential of the MixedVE as a memory training device, for all ages but especially for the older adults, which encourages us to continue this line of research. Aside from these applied implications, we developed a better understanding of the age differences in learning from a VE. Specifically, we know more about the effects of combined visualization design choices (realism levels with landmark locations) on the recall accuracy, confidence and visualization preferences of people from two distinctly different age groups in route recall tasks.

## Methods

We conducted a controlled experiment with a mixed factorial (2 × 2 × 3) design. *Age* was a between-subject factor (younger vs. older), *visualization type* (i.e., Abstract, Mixed, Realistic VEs), and *recall stage* (i.e., immediate vs. delayed) were within-subject factors. All participants performed route learning tasks in all three VEs and at two stages one week apart. As dependent variables, we measured the *recall accuracy* in all the tasks, with a focus on the direction of turns at intersection points, where we also measured participants’ *confidence* in their responses, and their visualization *preferences*.

### Participants

In total, 81 participants took part in the study: 42 in the younger group (27 ± 2 yrs., 23 female), and 39 in the older group (70 ± 4 yrs., 17 female). The younger participants were between 20–30 years of age and were recruited by word of mouth. The older participants were between 65–75 years of age and were recruited using the participant pool of UZH’s University Research Priority Program “Dynamics of Healthy Aging” (http://www.dynage.uzh.ch/en.html). This experiment was approved by the Ethical Committee of the Philosophical Faculty - University of Zurich with the form “Checkliste für die Selbstbeurteilung von Studien auf ethische Unbedenklichkeit”. All methods were performed in accordance with the relevant guidelines and regulations and all participants received informed consent, which after agreement they signed. All participants volunteered to participate, signed a written consent form and could withdraw their participation at any time. All participants performed the Mini-Mental State Examination to measure their cognitive status (MMSE). They were included in the study only if they scored a minimum of 27 out of 30^82^.

## Materials

### Apparatus

The experiment was performed in the 3D visualization/virtual reality lab of the GIVA unit of the Department of Geography, of the University of Zurich. Passive drive-throughs of the routes were presented as videos to participants on a large projection screen (230 × 140 cm). The participants were seated at a distance of 2.2 m from the screen to ensure that they could see the whole scene.

### Stimuli

Participants were shown videos of drive-throughs in a virtual fictitious city. Using procedural modeling, we designed the city to look as homogenous as possible to control for salient elements which might potentially interfere with route learning. Thus, the city contained buildings and other structures similar in size and architectural style, similar street network (intersection points with ~90 degree angles) over the whole city, and other visual elements (e.g., trees) were also kept similar to each other in size and other visual characteristics. We manipulated the design to obtain three different virtual environments (VEs, visualizations), as illustrated in Fig. 1. These three VEs differed in their degree of realism, and they represent the three main experimental conditions:

- a plain grayscale VE without any photo-textures (AbstractVE)
- a color photo-textured VE (RealisticVE)
- a mix of the two above, in which the buildings at all decision points towards the direction of the turn, and the structural network (street floors) are textured using color photography (MixedVE). Thus, we used photo-textures as a particular *type* of highlighting choice, because we work with realism as an important concept in route learning for transferability of acquired knowledge to the real world; and the *position* of the highlighted landmarks were selected based on landmark theories (we selected the positions that were previously shown as important positions where people took mental notes).

We created two routes in each of these three VEs. Each route consisted of seven intersections (three left, three right turns and one straight). The videos of the drive-through of these two routes were recorded at the same eye-level (1.50 m), had the same duration (100 sec) and were played back at the same speed (30 km/h). Each participant experienced two videos in all three VEs, adding up to a total of six different videos. Videos of the routes were shown only once. Using a Latin squares approach; we systematically rotated the order of the videos.

### Task

Participants were instructed to memorize the routes to the best of their ability. Once they watched the video, participants performed a series of different tasks as follows:

1. Identifying if a scene (screenshot) was on their route (“yes” or “no” answer), based on six screenshots from each path in each VE type (three were correct and three false). We call this set “visual tasks” as the participants would predominantly rely on visual information, while the location of the information was not relevant.
2. Drawing a sketch of the route using top-down screenshots of each VE. We called this set of tasks “spatial tasks”, because location and orientation are the key to solving the task, while the visual information is not as important.
3. Identifying the direction of turn at each of the seven intersection points based on a screenshot of the intersection point. We called this set of tasks “visuospatial tasks” one has to make use of visual cues, as well as location and orientation to solve the task. In other words, based on previous work^67,72–74,83^, we believed that participants would have to rely on both visual and spatial memory to solve this task. We thus considered this predominantly a visuospatial memory task.

More specifically, because participants responded to questions based on three VEs, for two routes in each VE, with a total of seven intersections at each video, they provided a total of 42 individual responses (3 × 2 × 7) in this task set. As mentioned earlier, the intersection points were presented as screenshots from the videos in a randomized order in the recall phase. Participants were asked to choose the direction in which they continued their route among the given options. Approximately one week after the first session (immediate recall stage), participants repeated the tasks without watching the videos again (delayed recall stage). Besides the “I don’t know” option, participants could mark *left*, *straight*, and *right*; giving them a 33% chance to guess the correct answer.

## Procedure

Upon arrival at the lab, participants signed an informed consent form. We briefed them about the procedure, introduced them to the hardware setup, and answered their questions, if they had any. We then assigned each participant to one of six videos (3 visualization types, 2 routes each). Before starting the actual route learning experiment, we showed participants a representative screenshot from each of the three VEs, and asked them to rate their preferences for a hypothetical route learning task. Immediately after this, the main experiment begun. Participants were given a scenario in which someone took them to a market in an unfamiliar neighborhood, and they were told to memorize the route as they would have to navigate the same route later by themselves. After watching each video only once, they answered a set of recall questions based on this specific video, and rated their confidence for each of their responses using a 5-point Likert scale that varied from “Not at all confident (1)” to “Very confident (5)”. After solving the tasks with three of the videos, participants could take a short break for approximately three minutes to counter potential fatigue. The last three videos followed in the same fashion. We then asked the participants which of the three VEs they preferred. There were no time limits in the experiment, thus the experimental duration of the first session (immediate recall stage) varied from 1 h to 1h40min. Participants came back six to eight days after the first session for the second session (delayed recall stage). In this stage, participants were not shown the videos again, thus they responded to the questions based on what they could recall from the first session. The duration of the second session varied from 40min to 1 h.

## Appendix: Additional analysis

### Spatial task

**Figure 7:**
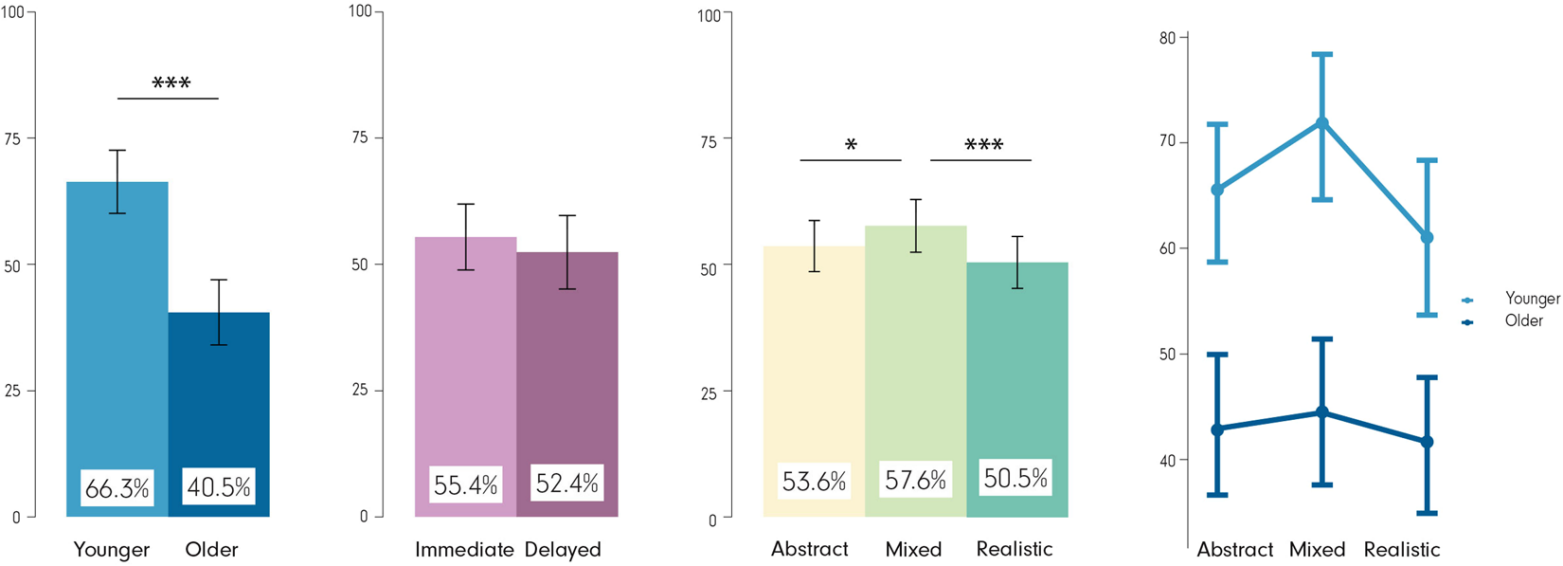
Spatial tasks. Main effects of (**a**) age, (**b**) recall stage, (**c**) visualization type on sketch task, and (**d**) interactions between age × visualization type (irrespective of recall stage). ***p < 0.001, *p < 0.05. Error bars: SEM.

A 2 (age) × 2 (recall stage) × 3 (visualization) mixed-design ANOVA revealed significant differences in the sketching task for two out of the three independent variables (no difference for recall stage). Figure 7 depicts the descriptive and inferential statistics; statistically significant differences were observed for (a) age F(1, 79) = 17.04, p < 0.001, ${{\rm{\eta }}}_{{\rm{p}}}^{2}$ = 0.15 (young: 66.3% ± 31.7%, older: 40.5% ± 30.0%), (b) visualization F(2, 158) = 11.69, p < 0.001, ${{\rm{\eta }}}_{{\rm{p}}}^{2}$ = 0.01 (Abstract: 53.6% ± 32.9%, Mixed: 57.6% ± 33.9%, Realistic: 50.5% ± 33.4%) and (c) age × visualization F(2, 158) = 3.80, p < 0.05, ${{\rm{\eta }}}_{{\rm{p}}}^{2}$ = 0.01. This interaction was driven by the significantly larger difference in the sketching performance between the Mixed and the Realistic visualizations for the younger participants compared to that of the older (young: 11.0% ± 21.3%, older: 3.0% ± 15.0%, t(149.35) = 2.77, p < 0.01, r = 0.22). Interestingly, the *recall stage* did not reveal statistically significant differences (immediate: 55.4% ± 32.1%, delayed: 52.4% ± 34.7%), neither did any other of the interactions.

Overall, the results from this task are in line with the visuospatial task. Age and visualization seem to matter for the performance, with the MixedVE resulting in best performance compared to both the Abstract and the Realistic VEs. Participants’ performance in the different recall stages was not significantly different in the spatial task. This might be explained by the active involvement required to fulfill the task. That is, the fact that participants actively *drew* the path immediately after the experiment, may have resulted in them learning to solve this task better than the other tasks in which they only passively watched the stimuli.

### Visual task

**Figure 8:**
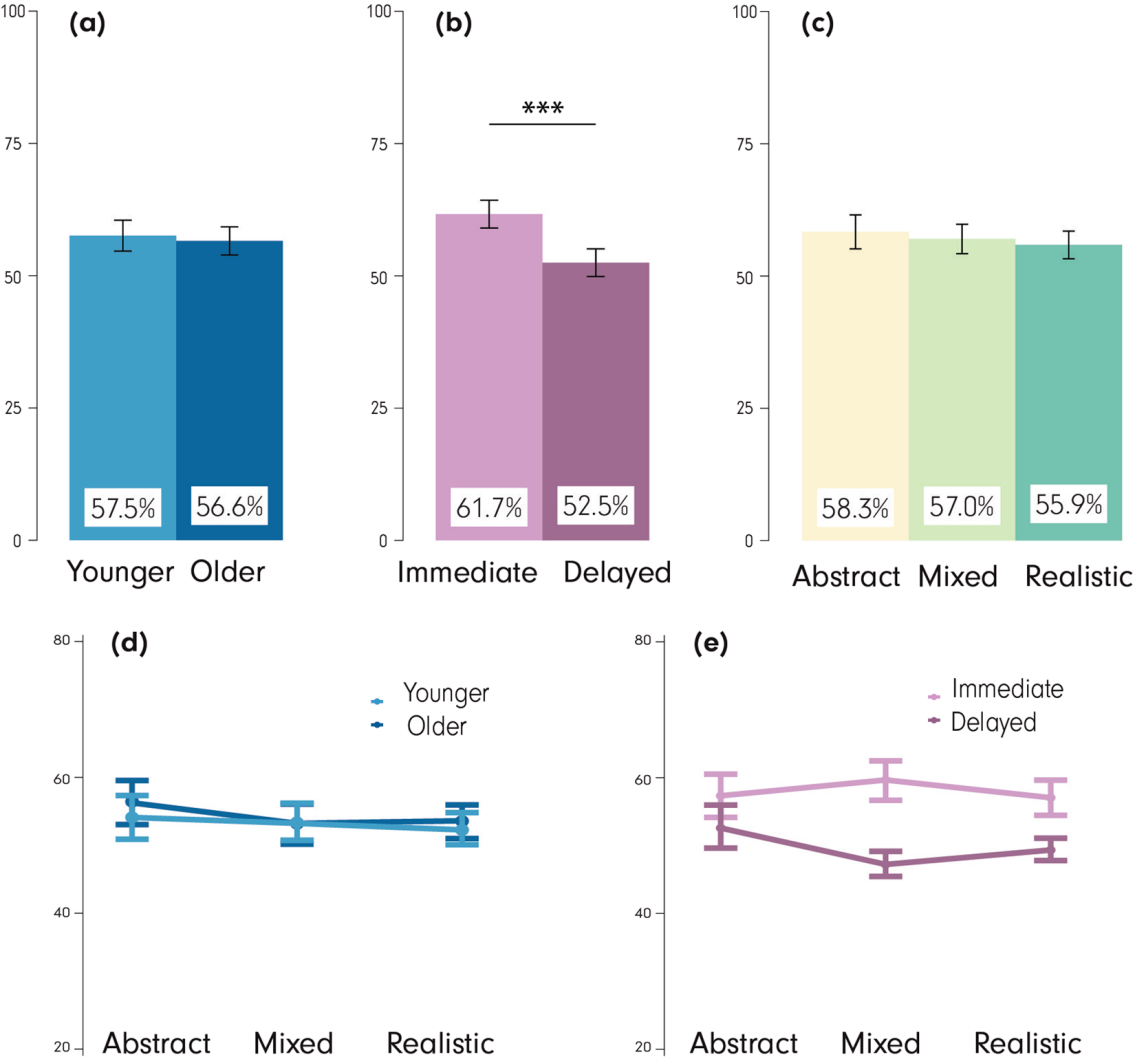
Visual tasks. Main effects of (**a**) age, (**b**) recall stage, (**c**) visualization type on visual task, and interactions between (**d**) age × visualization type (irrespective of recall stage), and (**e**) recall stage × visualization type (irrespective of age). ***p < 0.001. Error bars: SEM.

A 2 (age) × 2 (recall stage) × 3 (visualization) mixed-design ANOVA revealed significant differences in the visual task for only one of the three independent variables (no differences in age and visualization type, Fig. 8). Statistically significant differences were observed for *recall stage*, F(1, 79) = 39.71, p < 0.001, ${{\rm{\eta }}}_{{\rm{p}}}^{2}$ = 0.06 (immediate: 61.7% ± 17.0%, delayed: 52.5% ± 19.1%). Of the interactions, *age* × *visualization*, (F(2, 158) = 3.80, p < 0.05, ${{\rm{\eta }}}_{{\rm{p}}}^{2}$ = 0.01) and *recall stage* × *visualization interaction*, (F(2, 158) = 10.05, p < 0.001, ${{\rm{\eta }}}_{{\rm{p}}}^{2}$ = 0.03) rendered significant results. The *age* × *visualization* interaction was driven by the significantly larger difference in visual recall performance between the Mixed with the Abstract visualization for the younger participants compared to that of the older (young: 3.6% ± 26.8%, older: −6.7% ± 23.7%, t(159.65) = 2.59, p < 0.05, r = 0.20 [note the (−) in the older recall performance signifies higher recall for the Abstract compared to the MixedVE]). The *recall* × *visualization* interaction was driven by the significantly larger differences in the visual task performance between the Mixed and the Abstract visualization in the immediate compared to that of the delayed recall (immediate: 5.8% ± 20.7%, delayed: −8.5% ± 28.4%, t(146.32) = 3.66 , p < 0.001, r = 0.29), and the Abstract with the Realistic visualization (immediate: −3.2% ± 19.7%, delayed: 8.2% ± 26.2%, t(148.57) = 3.13 , p < 0.01, r = 0.25). Interestingly, *age* (young: 57.5% ± 19.5%, older: 56.6% ± 17.7%) and *visualization* (Abstract: 58.3% ± 20.8%, Mixed: 57.0% ± 18.0%, Realistic: 55.9% ± 16.9%) did not reveal statistically significant differences.

Note that regarding the age differences, the literature suggests that spatial memory functioning tends to decline with age, but visual memory might be ‘spared’^84^. Therefore, we have speculated from the start that the results for the visual task would be different than other memory tasks, which load on spatial memory more heavily. Initially, the results from the visual task do not seem to agree with the results from the visuospatial and the spatial tasks. Especially the interaction between *age* × *visualization* shows a conflicting pattern for the two age groups, with the MixedVE being more supportive for the young but not for the older, who seem to achieve higher recall with the AbstractVE. When examining the exact performance values from the visual task, however, we see that the performance is close to the chance level (50%) for the older participants. Thus, lack of interactions in this case may be due to task difficulty, which likely caused a “floor effect” for the older group and for both age groups in the delayed recall stage. In other words, overall task difficulty could have overshadowed these interactions.

### Data availability

The datasets generated during and/or analysed during the current study are available from the corresponding author on reasonable request.

## References

[1] Wolbers T, Dudchenko PA, Wood ER (2014). Spatial memory-a unique window into healthy and pathological aging. Front. Aging Neurosci..

[2] Lester AW, Moffat SD, Wiener JM, Barnes CA, Wolbers T (2017). The Aging Navigational System. Neuron.

[3] Klippel, A. *et al*. Direction concepts in wayfinding assistance systems. *Work*. *Artif*. *Intell*. *Mob*. *Syst*., 1–8 (2004).

[4] Münzer S, Zimmer HD, Baus J (2012). Navigation assistance: A trade-off between wayfinding support and configural learning support. J. Exp. Psychol. Appl..

[5] Parush A, Berman D (2004). Navigation and Orientation in 3D user Interfaces: The Impact of Navigation Aids and Landmarks. Int. J. Hum. Comput. Stud..

[6] Hultsch DF, Hertzog C, Small BJ, Dixon RA (1999). Use it or lose it: Engaged lifestyle as a buffer of cognitive decline in aging?. Psychol. Aging.

[7] Moffat S, Zonderman AB, Resnick SM (2001). Age differences in spatial memory in a virtual environment navigation task. Neurobiol. Aging.

[8] Muffato V, Della Giustina M, Meneghetti C, De Beni R (2015). Age-related differences in pointing accuracy in familiar and unfamiliar environments. Cogn. Process..

[9] Uttal DH (2013). The malleability of spatial skills: A meta-analysis of training studies. Psychol. Bull..

[10] Mitolo M, Borella E, Meneghetti C, Carbone E, Pazzaglia F (2017). How to enhance route learning and visuo-spatial working memory in aging: a training for residential care home residents. Aging Ment. Health.

[11] Lövdén M (2012). Spatial navigation training protects the hippocampus against age-related changes during early and late adulthood. Neurobiol. Aging.

[12] Hötting K, Holzschneider K, Stenzel A, Wolbers T, Röder B (2013). Effects of a cognitive training on spatial learning and associated functional brain activations. BMC Neurosci..

[13] Martin, M., Clare, L., Altgassen, A. M., Cameron, M. H. & Zehnder, F. Cognition-based interventions for healthy older people and people with mild cognitive impairment. *The Cochrane Library*, 10.1002/14651858.CD006220.pub2 (2011). 10.1002/14651858.CD006220.pub2 21249675

[14] Verhaeghen P, Marcoen A, Goossens L (1992). Improving memory performance in the aged through mnemonic training: A meta-analytic study. Psychol. Aging.

[15] Zehnder F, Martin M, Altgassen M, Clare L (2009). Memory training effects in old age as markers of plasticity: A meta-analysis. Restor. Neurol. Neurosci..

[16] Gross AL (2012). Memory training interventions for older adults: A meta-analysis. Aging Ment. Health.

[17] Richardson AE, Montello DR, Hegarty M (1999). Spatial knowledge acquisition from maps and from navigation in real and virtual environments. Mem. Cogn..

[18] Waller D, Bachmann E, Hodgson E, Beall A (2007). The HIVE: A huge immersive virtual environment for research in spatial cognition. Behav. Res. Methods.

[19] Jimeno A, Puerta A (2007). State of the art of the virtual reality applied to design and manufacturing processes. Int. J. Adv. Manuf. Technol..

[20] Bronfenbrenner U (1977). Toward an experimental ecology of human development. Am. Psychol..

[21] Optale G (2010). Controlling Memory Impairment in Elderly Adults Using Virtual Reality Memory Training: A Randomized Controlled Pilot Study. Neurorehabil. Neural Repair.

[22] Borkin MA (2013). What Makes a Visualization Memorable. IEEE Trans. Vis. Comput. Graph..

[23] Christou CG, Bülthoff HH (1999). View Dependence in Scene Recognition after Active Learning. Mem. Cognit..

[24] Meijer F, Geudeke BL, van den Broek EL (2009). Navigating through Virtual Environments: Visual Realism Improves Spatial Cognition. Cyberpsychology Behav..

[25] Smallman HS, John MS (2005). Naive Realism: Misplaced Faith in Realistic Displays. Ergon. Des..

[26] Wilkening, J. & Fabrikant, S. I. How Do Decision Time and Realism Affect Map-Based Decision Making? In *International Conference on Spatial Information Theory* 1–19, 10.1007/978-3-642-23196-4_1 (2011).

[27] Çöltekin, A., Francelet, R., Richter, K. F., Thoresen, J. & Fabrikant, S. I. The effects of visual realism, spatial abilities, and competition on performance in map-based route learning in men. *Cartogr*. *Geogr*. *Inf*. *Sci*. **45**, 10.1080/15230406.2017.1344569 (2017).

[28] Smallman HS, John M (2005). Naive realism: Limits of realism as a display principle. Proc. Hum. Factors Ergon. Soc. Annu. Meet..

[29] Salthouse T, Mitchell D, Skovronek E, Babcock R (1989). Effects of adult age and working memory on reasoning and spatial abilities. J. Exp. Psychol. Learn. Mem. Cogn..

[30] Kirasic KC (1991). Spatial cognition and behavior in young and elderly adults: implications for learning new environments. Psychol. Aging.

[31] Burns, P. C. Navigation and the Mobility of Older Drivers. **54**, 49–55 (1999). 10.1093/geronb/54b.1.s49 9934402

[32] Cherry KE, Park DC (1993). Individual difference and contextual variables influence spatial memory in younger and older adults. Psychol. Aging.

[33] Park DC (2002). Models of Visuospatial and Verbal Memory Across the Adult Life Span. Psychol. Aging.

[34] Nemmi F, Boccia M, Guariglia C (2017). Does aging affect the formation of new topographical memories? Evidence from an extensive spatial training. Aging, Neuropsychol. Cogn..

[35] Evans GW, Pezdek K (1980). Cognitive mapping: knowledge of real-world distance and location information. J. Exp. Psychol. Hum. Learn..

[36] Moffat SD, Resnick SM (2002). Effects of age on virtual environment place navigation and allocentric cognitive mapping. Behav. Neurosci..

[37] Muffato V, Meneghetti C, Di Ruocco V, De Beni R (2017). When young and older adults learn a map: The influence of individual visuo-spatial factors. Learn. Individ. Differ..

[38] Gyselinck V (2013). Considering spatial ability in virtual route learning in early aging. Cogn. Process..

[39] Dodson CS, Bawa S, Slotnick SD (2007). Aging, source memory, and misrecollections. J. Exp. Psychol. Learn. Mem. Cogn..

[40] Zelinski EM, Light LL (1988). Young and older adults’ use of context in spatial memory. Psychol. Aging.

[41] Dodson CS, Koutstaal W, Schacter DL (2000). Escape from illusion: Reducing false memories. Trends Cogn. Sci..

[42] Kelley CM, Sahakyan L (2003). Memory, monitoring, and control in the attainment of memory accuracy. J. Mem. Lang..

[43] Dodson CS, Bawa S, Krueger LE (2007). Aging, metamemory, and high-confidence errors: A misrecollection account. Psychol. Aging.

[44] Pardilla-Delgado, E. & Payne, J. D. The Deese-Roediger-McDermott (DRM) Task: A Simple Cognitive Paradigm to Investigate False Memories in the Laboratory. *J*. *Vis*. *Exp*., 1–10, 10.3791/54793 (2017). 10.3791/54793 PMC5407674 28190038

[45] Koutstaal, W. & Schacter, D. L. Gist-Based False Recognition of Pictures in Older and Younger Adults. **37**, 555–583 (1997).

[46] Dodson CS, Krueger LE (2006). I misremember it well: why older adults are unreliable eyewitnesses. Psychon. Bull. Rev. Rev..

[47] Zhong JY, Moffat SD (2016). Age-Related Differences in Associative Learning of Landmarks and Heading Directions in a Virtual Navigation Task. Front. Aging Neurosci..

[48] Estes, W. K. In *Handbook of Learning and Cognitive Processes* **5**, 271–295 (2014).

[49] Hegarty M (2004). Dynamic visualizations and learning: Getting to the difficult questions. Learn. Instr..

[50] Kirschner PA (2002). Cognitive Load Theory: Implications of Cognitive Load Theory on the Design of Learning. Learing Instr..

[51] Schnotz W (2002). Towards an integrated view of learning from text and visual displays. Educ. Psychol. Rev..

[52] Lokka, I. E. & Çöltekin, A. Remembering what we see: Designing virtual environments to improve visuo-spatial recall for navigation tasks. In *Proceedings of the 28th International Cartographic Conference* (2017).

[53] Lokka, I. E. & Çöltekin, A. Toward optimizing the design of virtual environments for route learning: empirically assessing the effects of changing levels of realism on memory. *Int*. *J*. *Digit*. *Earth*, 1–19 (2017).

[54] Waller D, Lippa Y (2007). Landmarks as beacons and associative cues: Their role in route learning. Mem. Cognit..

[55] Lenneberg EH (1961). Color Naming, Color Recognition, Color Discrimination: A Re-Appraisal. Percept. Mot. Skills.

[56] Mayer RE, Moreno R (2003). Nine Ways to Reduce Cognitive Load in Multimedia Learning. Educ. Psychol..

[57] Çöltekin, A., Bleisch, S., Andrienko, G. & Dykes, J. Persistent challenges in geovisualization – a community perspective. *Int*. *J*. *Cartogr*. **3**, 1–25 (2017).

[58] Yerkes RM, Dodson JD (1908). The relation of strength of stimulus to rapidity of habit-formation. J. Comp. Neurol. Psychol..

[59] Röser, F., Krumnack, A., Hamburger, K. & Knauff, M. A four factor model of landmark salience – A new approach. *Proc*. *11th Int*. *Conf*. *Cogn*. *Model*. 82–87 (2012).

[60] Röser F, Hamburger K, Krumnack A, Knauff M (2012). The Structural Salience of Landmarks: Results from an On-Line Study and a Virtual Environment Experiment. J. Spat. Sci..

[61] Karimpur, H., Röser, F. & Hamburger, K.Finding the Return Path: Landmark Position Effects and the Influence of Perspective. F*ront*. *Psychol*. 7 (2016). 10.3389/fpsyg.2016.01956 PMC5180192 28066283

[62] Winter, S. Route Adaptive Selection of Salient Features. In *International Conference on Spatial Information Theory* 349–361, 10.1007/978-3-540-39923-0_23 (2003).

[63] Richter, K.-F. & Winter, S. *Landmarks*, 10.1007/978-3-319-05732-3 (2014).

[64] Raubal, M. & Winter, S. Enriching Wayfinding Instructions with Local Landmarks. *Proc*. *Second Int*. *Conf*. *Geogr*. *Inf*. *Sci*., 243–259 (2002).

[65] Klippel, A. & Winter, S. Structural Salience of Landmarks for Route Directions. In *International Conference on Spatial Information Theory* (ed. Springer Berlin Heidelberg), 347–362, 10.1007/11556114_22 (2005).

[66] Waller D, Greenauer N (2007). The role of body-based sensory information in the acquisition of enduring spatial representations. Psychol. Res..

[67] Wiener JM, de Condappa O, Harris MA, Wolbers T (2013). Maladaptive bias for extrahippocampal navigation strategies in aging humans. J. Neurosci..

[68] Strickrodt, M., O’Malley, M. & Wiener, J. M. This Place Looks Familiar — How Navigators Distinguish Places with Ambiguous Landmark Objects When Learning Novel Routes. *Front*. *Psychol*. **6** (2015). 10.3389/fpsyg.2015.01936 PMC4689859 26733921

[69] Aginsky V, Harris C, Rensink R, Beusmans J (1997). Two strategies for learning a route in a driving simulator. J. Environ. Psychol..

[70] Claramunt C, Winter S (2007). Structural Salience of Elements of the City. Environ. Plan. B Plan. Des..

[71] Merriman, N. A. *et al*. Crowded environments reduce spatial memory in older but not younger adults. *Psychol*. *Res*. **82**, 1–22, 10.1007/s00426-016-0819-5 (2016). 10.1007/s00426-016-0819-5 27783147

[72] Rosenbaum, R. S., Winocur, G., Binns, M. A. & Moscovitch, M. Remote spatial memory in aging: all is not lost. *Front Aging Neurosci.* **4**, 1–10 (2012). 10.3389/fnagi.2012.00025 PMC3440628 22993506

[73] Moffat SD, Elkins W, Resnick SM (2006). Age differences in the neural systems supporting human allocentric spatial navigation. Neurobiol. Aging.

[74] Wiener JM, Kmecova H, de Condappa O (2012). Route repetition and route retracing: Effects of cognitive aging. Front. Aging Neurosci..

[75] Janzen G (2006). Memory for object location and route direction in virtual large-scale space. Q. J. Exp. Psychol..

[76] Charness N (1981). Visual short-term memory and aging in chess players. J. Gerontol..

[77] Fandakova Y (2018). Age differences in false memory: The importance of retrieval monitoring processes and their modulation by memory quality. Psychol. Aging.

[78] MacDonald SWS, Stigsdotter-Neely A, Derwinger A, Backman L (2006). Rate of acquisition, adult age, and basic cognitive abilities predict forgetting: New views on a classic problem. J. Exp. Psychol. Gen..

[79] Old SR, Naveh-Benjamin M (2008). Differential Effects of Age on Item and Associative Measures of Memory: A Meta-Analysis. Psychol. Aging.

[80] Sporer SL, Penrod S, Read D, Cutler B (1995). Choosing, confidence, and accuracy: A meta-analysis of the confidence-accuracy relation in eyewitness identification studies. Psychol. Bull..

[81] Smallman HS, Cook MB (2011). Naïve Realism: Folk fallacies in the design and use of visual displays. Top. Cogn. Sci..

[82] O’Bryant SE (2008). Detecting Dementia with the Mini-Mental State Examination (MMSE) in Highly Educated Individuals. NIH Public Access.

[83] Sullivan, C. O. & Newell, F. N. Crowded environments reduce spatial memory in older but not younger adults. *Psychological research* **82**, 407–428, 10.1007/s00426-016-0819-5 (2016). 10.1007/s00426-016-0819-5 27783147

[84] Sekuler R, Kahana MJ, McLaughlin C, Golomb J, Wingfield A (2005). Preservation of Episodic Visual Recognition Memory in Aging. Exp. Aging Res..

